# Functional Biomaterials: Scaffolds for Innovative Treatments

**DOI:** 10.3390/jfb16050154

**Published:** 2025-04-27

**Authors:** Cristian Scheau, Andreea Cristiana Didilescu, Constantin Caruntu

**Affiliations:** 1 Department of Physiology, The “Carol Davila” University of Medicine and Pharmacy, 050474 Bucharest, Romania; cristian.scheau@umfcd.ro (C.S.); costin.caruntu@gmail.com (C.C.); 2 Department of Radiology and Medical Imaging, “Foisor” Clinical Hospital of Orthopaedics, Traumatology and Osteoarticular TB, 021382 Bucharest, Romania; 3 Department of Embryology and Microbiology, Faculty of Dentistry, The “Carol Davila” University of Medicine and Pharmacy, 050474 Bucharest, Romania; 4 Department of Dermatology, “Prof. N.C. Paulescu” National Institute of Diabetes, Nutrition and Metabolic Diseases, 011233 Bucharest, Romania

Numerous therapies for a multitude of pathologies have reached their limits [1]. Although disease management and medical care are standardized, we must acknowledge that each patient is different, with varied responses to the disease and the cure [2]. Moreover, for many diseases, the treatment depends on the ability of the medical system to provide donor organs, the availability of which is highly limited [3]. Some patients do not respond well to non-targeted treatments, whether for cancer or in cases where low-specificity drugs are utilized [4]. Thus, it is essential to identify options for specific biocompatible transporters with high fidelity for certain affected areas. These are only a few of the major problems for which the accelerated development of biomaterials and manufacturing technologies in recent years has brought promising solutions [5,6].

One area where many biomaterials have shown excellent results is orthopedics. However, the 3D printing of these materials poses challenges regarding degradation over time and compatibility with the human body, especially at the level of the graft–real tissue interface [7]. Enthusiastic research conducted using viable cells in combination with biomaterials has already tested the mechanical stability and bioactive properties of these materials, yielding great results in this field [8].

In their comprehensive review, Timofticiuc et al. explained how the properties of 3D-bioprinted constructs can spare patients from limb amputation [9]. Surgical teams with a strong vascular microsurgery background have perfectly integrated full or partial cell-laden biomaterial-based bones as replacements for bones that were candidates for amputation due to severe defects. Full bone grafts employed in minor patients and the designs used for optimally anchoring the 3D-bioprinted constructs with the surrounding tissues constitute some of the true innovations. Newly developed biomaterials situated in the testing area either in vitro or in animal models, the printing techniques used, and a summary of their physical and chemical properties are also presented in this review paper [9].

Designing large bone grafts is a challenging task, mainly because of the poor blood supply, which limits the exchange of metabolites and the supply of oxygen and nutrients [10,11]. Integrating viable cells into the grafts offers great advantages regarding their biocompatibility but also creates residues, which accumulate and precipitate the development of a cytotoxic microenvironment in the absence of proper blood flow [12]. A potential solution to this issue is designing pre-vascularized grafts, a method exemplified by Buranawat et al. in their research on maxillofacial reconstruction [13]. The team designed a porous calcium metaphosphate scaffold in which a co-culture containing endothelial cells and human alveolar osteoblasts was introduced. Creating porous structures is essential for cell development, especially in bone structures, where osteointegration, osteoconductivity, and angiogenesis are critical for graft sustainability [14,15]. The pores created in this study ranged from 80 to 100 µm and were optimal for cell development, which resulted in optimal intrinsic VEGF-release (10,455.6 pg/mL), the main factor for angiogenesis. Furthermore, a scaffold with rapid biodegradability was created using calcium metaphosphate, a bioactive ceramic that retains large quantities of water. This property, although potentially affecting the mechanical strength of the grafts, allows enhanced tissue integration and cell viability. Hence, a pre-vascularized graft was obtained, thus overcoming a major drawback of large bone grafts [13].

Some orthopedic grafts are designed in a way that prioritizes elasticity rather than mechanical strength [16]. In the realm of adult-acquired flatfoot deformities, a spring ligament (SL) injury requires the replacement of a ligament with a synthetic graft, which should be bioactive and possess essential mechanical properties, such as elasticity and stress resistance [17]. In their research, Nieto et al. designed an SL graft made of polycaprolactone (PCL) and type-B gelatin enhanced with graphene oxide. The graft was tested in vitro to demonstrate its bioactive properties and later studied in silico regarding its biomechanical behavior. PCL and gelatin were chosen for their known biocompatibility and optimal wettability as well as their ability to promote cell adhesion and proliferation for better in situ integration. To create a ligament structure, a fiber-like structure was obtained using electrospinning. However, the nanofibers resulting from using this technique lacked mechanical strength, an issue addressed by adding nanocomposites such as graphene oxide. Finding the optimal fiber concentration and orientation is essential for a precise ligament equivalent. In this study, the optimal Young’s Modulus of 240 MPa was achieved with a 2% graphene oxide concentration in a twisted yarn configuration of the ligament graft [17].

There are also cases where the mechanical properties of biomaterials are not as relevant to a given task as they are for the majority of the pathologies for which they are used. A good example is provided in the study by Tabanella et al., who designed a collagen scaffold for tissue augmentation. The aesthetic aspect of a repair is a major concern in the realm of oral defects, and mucosal height plays a crucial role in this regard [18]. In a comparison between porcine-cross-linked collagen and a traditional approach for vertical augmentation of the oral mucosa, the former was demonstrated to be an aesthetic solution and statistically correlated with an enhanced height of the keratinized mucosa [19].

Another major issue in bone healing pertains to the low estrogen levels in menopausal women, who have high osseous tissue catabolism and pose difficulties in the restauration of peri-implant bone defects [20]. Duarte et al. managed to design a scaffold made of deproteinized bovine bone with genistein via sonification to mimic the local, peri-dental effects of estrogen and tested it in ovariectomized rats. Genistein is a phytoestrogen that can act on estrogen receptors in the surrounding bone, activating osteoblast differentiation [21]. To further test bone production and implant integration, the removal torque was calculated, and the genistein-loaded scaffold presented the highest value, 5.4 Ncm, as well as the highest bone volume/tissue volume compared to the test group, wherein no estrogen equivalents were used [20].

In the last decade, a new branch of industry rapidly developed alongside new biomaterials and methods for better integration. Finding new technologies with which to transform biomaterials from micro- to nano-dimensions and using them as carriers or coatings constitute new and exciting directions for research in the field of 3D printing and biomaterials [22,23]. Boyuklieva et al. developed microspheres made of nanocomposites for use as drug carriers [24]. Substances with very short biological half-lives require higher serum concentrations for long and effective responses, but these high concentrations may cause severe adverse reactions. Thus, by creating microspheres composed of PCL nanoparticles and controlling the pH levels, good vectors exhibiting tropism to hard-penetrating tissues were designed, and the effects of the drug were preserved, while the side effects were not [24].

Kaupbayeva et al.’s study helps us to further understand how biomaterials can be used as vectors not only for drugs but also in gene editing [25]. CRISPR/Cas9 is a gene-editing technique that presents many drawbacks, especially with respect to the intriguing area of genetic defects [26]. This comprehensive review presents the methods and tools used to create carriers, such as vectors, biomaterials, synthetic approaches, and physical methods. The technologies and techniques were reviewed and efficiently categorized in order to clarify the possibilities that have been discovered so far, highlighting the vast advantages that biomaterials can bring to the medical realm [25].

Another example of a nanoparticle application is presented in the research conducted by Melchor-Moncada et al., where a coating of titanium oxide nanoparticles was used to improve the stability and antibacterial activity of serratiopeptidases [27]. Serratiopeptidase is a natural bacterial enzymatic compound and an innovative alternative to antibiotics [28]. However, its low solubility, heightened sensitivity to temperature and pH variations, and self-cleavage tendency are some of the traits hindering its large-scale usage in the context of antibiotic resistance [28]. However, in the cited paper, the research team managed to achieve enzyme immobilization through bioconjugation with titanium oxide nanoparticles, increasing stability and doubling antibacterial activity, especially against E.coli [27].

Magnesium alloys also present good biocompatibility and may be used as implants. However, they are highly corrosive and do not perform well under dynamic stress [29]. Saqib et al. used plasma electrolytic oxidation to create an anodic layer on top of a magnesium alloy while also coating it with biopolymers, granting it excellent performance under dynamic stress without signs of corrosion [30]. However, the coatings eventually degraded, and the added layer disappeared almost completely after approximately 30 days, returning the implant to its original corrosion rate. These results are promising, especially for the few cases where stents are constructed from magnesium alloys, but further studies must be conducted to improve the survival rates of the implants and develop additional applications for these metal alloys [30].

Biomaterials also present numerous advantages in cancer research and treatment. The paper by Knight et al. highlights the piezoelectric properties of nanomaterials and how said properties can be used to efficiently treat glioblastoma, with reduced side effects [31]. Many novel treatments for glioblastoma involve the use of electric fields to target cancerous cells, but such methods carry a risk of serious burns [32]. By introducing nanomaterials to the cancer site and applying a mechanical force such as ultrasound, biomaterials can exhibit piezoelectric properties and generate a local electric field that stops the tumoral cells from proliferating [31]. In this review, a classification of all the organic or inorganic nanomaterials that can exhibit these properties is presented, highlighting the technologies used and their properties in combating tumoral cells [31].

Biomaterials can also be used in research on tumoral cell activity. Building 3D models that mimic the microenvironments of different cancers is a novel approach to gaining further insight into the development and behavior of tumor cells. Evangelista et al. presented the techniques and models used in this direction for head-and-neck squamous cell carcinoma [33]. They also highlighted how the mechanical properties of these 3D-printed models translate the behaviors of tumoral cells. Stiffness, elasticity, and dynamic behavior are some of the properties that influence tumor growth, metastasis, and treatment response. Furthermore, understanding the type of mechanical environment required by cancer cells can contribute to conceptualizing a treatment. Altering cell tension, hydrostatic pressure, mechanical strength, Young’s modulus, or viscosity could play a role in the therapeutic approach [33].

The data presented in the latest studies demonstrate that the field of biomaterials is rapidly developing, with excellent results in numerous medical areas. Future research will be able to translate more characteristics of organs and tissues into scaffolds or other biomaterial constructs in order to mimic human structures and functionality to a greater extent. Furthermore, as biomaterials are incorporated into modern therapies, current treatments will likely improve, with better targeting and efficiency, while risks associated with human errors will be progressively mitigated.

## Data Availability

Not applicable.
